# Dissecting the non-neuronal cell contribution to Parkinson’s disease pathogenesis using induced pluripotent stem cells

**DOI:** 10.1007/s00018-020-03700-x

**Published:** 2020-11-18

**Authors:** Meritxell Pons-Espinal, Lucas Blasco-Agell, Antonella Consiglio

**Affiliations:** 1 Department of Pathology and Experimental Therapeutics, Bellvitge University Hospital-IDIBELL, 08908 Hospitalet de Llobregat, Spain; 2 Institute of Biomedicine (IBUB) of the University of Barcelona (UB), 08028 Barcelona, Spain; 3 Department of Molecular and Translational Medicine, University of Brescia, Piazza del Mercato, 15, 25121 Brescia, BS Italy

**Keywords:** Parkinson’s disease, iPSC, Neurodegeneration, Dopaminergic neurons, Glia, Disease modeling, Organoid

## Abstract

Parkinson’s disease (PD) is an incurable age-linked neurodegenerative disease with characteristic movement impairments that are caused by the progressive loss of dopamine-containing neurons (DAn) within the substantia nigra pars compacta. It has been suggested that misfolded protein aggregates together with neuroinflammation and glial reactivity, may impact nerve cell function, leading to neurodegeneration and diseases, such as PD. However, not many studies have been able to examine the role of human glial cells in the pathogenesis of PD. With the advent of induced pluripotent stem cell (iPSC) technology, it is now possible to reprogram human somatic cells to pluripotency and to generate viable human patient-specific DA neurons and glial cells, providing a tremendous opportunity for dissecting cellular and molecular pathological mechanisms occurring at early stages of PD. This reviews will report on recent work using human iPSC and 3D brain organoid models showing that iPSC technology can be used to recapitulate PD-relevant disease-associated phenotypes, including protein aggregation, cell death or loss of neurite complexity and deficient autophagic vacuoles clearance and focus on the recent co-culture systems that are revealing new insights into the complex interactions that occur between different brain cell types during neurodegeneration. Consequently, such advances are the key to improve our understanding of PD pathology and generate potential targets for new therapies aimed at curing PD patients.

## Introduction Parkinson’s disease

Parkinson’s disease (PD) is the most common neurodegenerative disease after Alzheimer’s disease (AD) affecting more than 6.5 million people worldwide [1], which represents 2–3% of the population over the age of 65 years [2, 3]. Clinically, PD is characterized by resting tremor, slowness of movement, rigidity, and postural instability, although non-motor symptoms are also important at different stages of the disease [4, 5]. Motor impairment of PD patients is mainly due to the insidious degeneration of dopaminergic neurons (DAn) of the substantia nigra pars compacta (SNc), leading to a reduction in the levels of dopamine in the striatum. This pathological process is also accompanied by the formation of cytosolic protein aggregates named Lewy bodies/neurites in surviving neurons, composed mainly of the α-synuclein protein (α-syn). Microglial activation and an increase in astroglia and lymphocyte infiltration also occur in PD [6–8].

Although the majority of PD cases (90–95%) are of unknown etiology, so-called idiopathic PD, around 5% have been shown to have a genetic basis, with mutations in the leucine-rich repeat kinase 2 (*LRRK2*) gene accounting for the largest number of patients of familial PD. Interestingly, LRRK2 polymorphisms are also considered a relevant genetic determinant for sporadic PD, and LRRK2 function appears dysregulated in sporadic cases of PD, even in the absence of LRRK2 mutations/polymorphisms. Pathogenic variants in α-syn (*SNCA*) gene have been also identified and associated to PD, while mutations in four genes (*Parkin, DJ-1, PINK1* and *ATP13A2*) cause early-onset parkinsonism [9].

Pharmacological substitution with the dopamine precursor levodopa (l-DOPA), dopamine agonists and anti-cholinergics or electrophysiological substitution after surgery [3] represent therapeutic options for PD patients that can reduce motor symptoms and partially improve their quality of life. However, so far, no existing therapies can cure or delay neuronal damage and disease progression. Further, long-term levodopa treatment leads to a gradual loss in restorative benefit, and most patients develop severe motor and psychiatric side effects, reducing the overall medication efficacy [9]. Thus, it is crucial to establish new therapies to possibly slow or reverse the neurodegenerative process itself. However, the unknown etiology of the idiopathic forms and the emerging view that non-neuronal cells could be also implicated in the pathophysiology of the disease, greatly impact on the development of accurate models and on the discovery of a definitive cure [10].

## Modeling PD using iPSC technology

The pathogenic mechanisms that lead to neurodegeneration in PD are not well understood, since current experimental PD models do not recapitulate key neuropathological features of the disease [11]. In particular, the special susceptibility of DAn to neurodegeneration and the progressive nature of this process in PD, together with the presence of Lewy bodies, have proven especially difficult to model in animal models of PD. For instance, genetically engineered animal models based on overexpression (driven by non-native or physiological human promoters) or knockout of the gene of interest only partially replicate key features of the neurodegeneration in PD [12]. Ideally, PD should be studied in patient cells, but the availability of living cells for functional studies is almost completely limited to neuronal tumor cell lines, which incompletely reflect the characteristics on non-dividing human DAn. For this reason, the bulk of human studies have been performed in frozen or fixed brain samples, allowing only for steady-state analysis.

A major advancement for modeling complex diseases, such as PD, overcoming the limitations mentioned above, came from the application of new technologies to reprogram fibroblasts into induced pluripotent stem cells (iPSC) [13–16]. As these cells, like human embryonic stem cells (hESC), are defined by their ability to undergo limitless self-renewal and to differentiate into any cell type of the three embryonic germ layers in vitro and in vivo, including central nervous system lineages, this technology provides for the first time an unlimited source of native phenotypes of cells specifically involved in neurodegeneration in PD in vitro. A key advantage of cell reprogramming is also the possibility of generating iPSC from patients that carry the precise genetic variants, both known and unknown, which may contribute to the disease. Importantly, they are isogenic to the donor individual since they carry the same genetic background, providing the unprecedented opportunity to recapitulate both normal and pathologic human tissue formation in vitro, and thereby facilitating disease investigation (such as functional relevance of the molecular findings, contribution of individual genetic variations, patient-specific response to specific interventions) and they also help to recapitulate the time-course of the disease and drug development. To date several groups including ours, have reported the derivation of iPSC from patients suffering sporadic and familial PD, which were used to generate patient-specific ventral midbrain dopaminergic neurons (vmDAn) that are the exact cell type that die in brain patients. Interestingly these studies have proven that key features of PD pathophysiology such as abnormal α-syn accumulation, alterations in the autophagy machinery, and increased susceptibility to undergo neurodegeneration, can be modeled in those vmDA neurons derived from PD-specific iPSC [17–20].

Importantly, Sánchez-Danés et al. studies provided the first proof-of-principle evidence that neurons with the genome of sporadic PD patients exhibited similar phenotypes as seen in iPSC derived from patients with monogenic PD [17]. Since earlier attempts by others at modeling PD through iPSC technology failed to identify such phenotypes [21, 22], most likely the success of this strategy depended on the ability to maintain DAn cultures over a long-term culture span, and the use of multiple patients per condition, which allowed controlling the inherent variability of human pluripotent stem cell lines. In addition, a number of studies have described the susceptibility of PD iPSC-derived DAn to cytotoxic agents and consequent cell death, and several works have also reported on the mechanisms underlying these processes, highlighting four clearly affected cellular pathways that convergence between different familial forms of PD (including mutations in *LRRK2, SNCA, PINK1, GBA1, PARK2* and *DJ-1*) and idiopathic patients: mitochondrial function and oxidative stress, autophagy-lysosomal metabolism, ubiquitin–proteasome protein degradation, and endoplasmic reticulum stress/unfolded protein response [17, 18, 23–32] (Table 1). However, as the complexity of sporadic PD is undefined, for the majority of PD cases a concerted interplay of environmental risk factors and specific genetic susceptibility factors, still needs to be modeled in vitro.

**Table 1:** Summary of most relevant studies using iPSC cells in PD

Cell type	Main achievement	Phenotype observed	References
Neuron	Efficient reprogramming of PD fibroblasts into iPSC and differentiation into DAn	No significant alterations	Soldner [21]
	Generation of isogenic PD iPSC lines using ZFN-mediated genome editing	No significant alterations	Soldner [22]
	Enhanced stress sensitivity and cell death of DAn upon exposure to stress agents	PD-DAn showed increased key oxidative stress-response genes and of α-syn protein, caspase 3 and cell death	Nguyen [18]
	Efficient modeling of PD-associated neurodegenerative alterations upon long-term dopaminergic cultures without addition of stress agents	PD-DAn showed aberrant neurite morphology, cell death and impaired autophagic clearance	Sánchez-Danés [17]
	Demonstration of convergence of cellular disease mechanisms between different familial forms of PD	PD-DAn showed oxidative stress and mitochondrial dysfunction	Cooper [23]
	Correction of LRRK2-G2019S mutation using target gene editing	Disease phenotypes were rescued by target correction of LRRK2-G2019S, or recapitulated after knock-in the mutation	Liu [35], Reinhardt [36]
	First scRNA-seq from iPSC-derived neurons	Identification of HDAC4 as a regulator of PD cell phenotypes	Lang [34]
Astrocyte	Efficient generation and characterization of iPSC-derived astrocytes from PD patients	Impaired macroautophagy and CMA leading to α-syn accumulation	Di Domenico [80]
Monocyte/macrophage	Generation of iPSC-derived monocytes from LRRK2-PD patients	Increased pro-inflammatory response and deficits in migration capacity in PD monocytes	Speidel [102]
	Generation of iPSC-derived macrophages from SNCA Triplication and SNCA A53T PD patients	Increased levels of intracellular α-syn, which compromised phagocytosis as determined by measuring fibrillar α-syn uptake	Haenseler, [100]
Interaction 2D co-cultures	Non-cell autonomous contribution of astrocytes to DAn neurodegeneration in a 2D co-culture system	PD astrocytes induce DAn neurodegeneration at least in part due to α-syn transfer	Di Domenico [80]
	Study the contribution of the adaptive immune system to neurodegeneration in a 2D co-culture system	Th17 lymphocytes induce neuronal cell death through IL-17/IL-17R signaling	Sommer [43]
3D midbrain organoids	Modeling PD using midbrain organoids	PD midbrain organoids recapitulate disease-relevant phenotypes including reduced number and complexity of DAn and deposits of thioflavin corresponding to α-syn	Smits [131], Kim [130]
Chimeric model	Preclinical study demonstrating the clinically applicable use of iPSC-derived dopaminergic progenitors for PD treatment	Transplanted human iPSC-derived dopaminergic progenitors into a primate PD model survived and functioned as DAn	Kikuchi [142]

*α-syn* α-synuclein, *CMA* chaperone-mediated autophagy, *DAn* dopaminergic neurons

Although the specific molecular mechanisms by which DAn from PD patients are more susceptible to degenerate are unknown, recent data identified a toxic cascade of mitochondrial and lysosomal dysfunction specific from human-derived PD neurons that was mediated by the accumulation of oxidized dopamine and α-syn. Remarkably, however, neither oxidized dopamine nor α-syn accumulation are found in PD mouse models. Therefore, the inherent species-specific differences between human and mouse neurons stresses the value of studying human neurons to identify relevant targets [33].

In the last few years, RNA-sequencing (RNA-seq) and gene editing techniques have been also used as new molecular tools to help dissect the specific mechanisms underlying DAn degeneration in the pathophysiology of PD. Indeed the use of high-resolution single-cell RNA-seq of iPSC-DAn, which avoids the confounding effects of heterogeneous and asynchronous neuronal cultures [34], is beginning to provide new insights into the molecular mechanisms of DAn degeneration. In the context of gene editing studies, Liu and colleagues were the first to correct the LRRK2-G2019S mutation in neural stem cells (NSC) derived from iPSC using a helper-dependent adenoviral vector and homologous recombination, and demonstrated nuclear envelope aberrations associated with this mutation [35]. Shortly after, Reinhardt and colleagues used zing finger nucleases (artificial restriction enzymes) to specifically correct the LRRK2-G2019S mutation in isogenic iPSC lines, and demonstrated a direct link between the mutation and axonal length and sensitivity to PD stressors [36]. More recently, Soldner and colleagues used CRISPR/Cas9-based gene editing to uncover the effect of a PD-related single-nucleotide polymorphism in the *SNCA* locus by generating a collection of isogenic lines, identifying a common PD-associated risk variant in a non-coding distal enhancer element that regulates the expression of α-syn by differential binding of two brain-specific transcription factors (EMX2 and NKX6-1) [37].

The generation of promoter-lineage reporter iPSC lines through gene editing tools has advanced on the identification of specific cell types from heterogeneous culture. Specifically, DAn lineage reporters, such as those based on the endogenous expression of tyrosine hydroxylase (TH) regulatory sequences, have been introduced using CRISPR/Cas9-based editing strategies, allowing the specific discrimination and visualization of DAn (TH+) cells in living cell cultures [38–40]. Moreover, these novel genetic TH reporter systems enable to isolate and purify DAn and later resume in vitro culture while preserving their dopaminergic identity. Thus, these new tools are likely to facilitate future research on the processes associated with specific DAn biology and disease help in dissecting the specific vulnerability of DAn in PD.

**Fig. 1:**
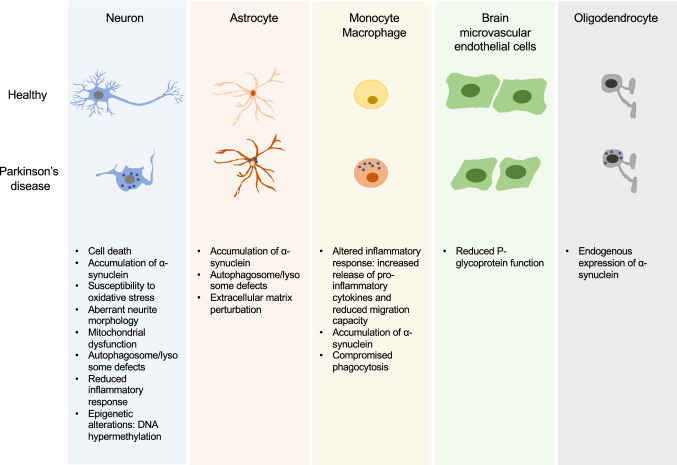
Summary of major phenotypic alterations from different brain cell types in PD

Given that there is an extensive debate on whether PD is a truly neuronal autonomous disease, iPSC technology which allows generating different brain cell types that might be implicated in PD, such as microglia and astrocytes, will contribute to dissect the genetic, age-related, and cell-type-specific factors that lead to PD (Fig. 1).

## Using iPSC-based models to test the contribution of non-neuronal cell types in Parkinson’s disease

For decades, research on PD has focused on understanding the mechanisms underlying the death of DAn from the SNc and α-syn accumulation [5, 9, 41]. However, although these distinct histological changes are well known, other concomitant pathological alterations, such as neuroinflammation and glial reactivity are increasingly gaining interest as they might sustain or exacerbate DAn degeneration [6, 42–44]. For instance, pro-inflammatory mediators are increased in the cerebrospinal fluid, serum and SNc at Braak stage 1 and 2 before α-syn appears [45, 46] and has been correlated with a worst PD prognosis [47, 48]. Moreover, microgliosis and reactive astrocytes have been found in PD postmortem studies and even more recently, activated microglia has been detected on in vivo PET imaging analysis of prodromal and diagnosed PD patients [42, 49, 50]. Microglia and astrocytes might be initially activated and recruited to create a protective environment around the damaged DAn [51, 52]. However, recent hypothesis suggests that during the course of the disease glial cells may switch to a toxic phenotype, as shown by reduced capability to maintain neuronal homeostasis, leading to an increase in cytokine overexpression/dysregulation, which accelerates neurodegeneration [42, 53, 54]. These altered reactive cell responses might be influenced by aging or by the presence of specific genetic mutations significantly linked to PD [42, 55].

Although the exact molecular mechanisms underlying neuroinflammatory processes in PD are not completely understood, members of the inflammasome, including NOD‐like receptor (NLR) family, have recently been linked to the pathogenesis of Alzheimer's disease (AD), and are suspected to contribute to neurodegeneration in PD, since they can potentially be activated by oxidative stress and insoluble α-syn aggregates [56–59]. Nevertheless, the contribution of inflammasome activation to driving α-syn pathology and DAn degeneration is still unclear.

Recently, different groups identified significant associations between risk loci and specific cell types. In this regard, by integrating genome-wide association study (GWAS) results from PD with single-cell transcriptomic data, it has been shown that PD could be genetically associated not only with cholinergic and monoaminergic neurons (which include DAn) but also with enteric neurons and oligodendrocytes [60]. In contrast, other studies identified significant associations with cell types from the adaptive immune system [61]. Previous results instead found no cell-type-specific association with PD but rather risk loci related with global cellular processes detectable across several cell types including microglia, astrocytes and oligodendrocytes [7]. Moreover, PD-related risk gene expression levels are similar between different brain cell types [62]. All together, these results support the view that PD is a disorder of global processes working across various cell types. In this respect, patient-specific iPSC would allow to recreate PD in a dish to uncover disease drivers and mechanisms. Successful efforts in this regard, via generating different CNS and non-CNS cell types as well as 2D and 3D co-culture systems, will be discussed below.

## Human iPSC-derived astrocytes

Astrocytes are the largest glial cell population in the central nervous system (CNS) and possess several key features including their interaction with critical functional–anatomical domains in brain regions, which can potentially influence neurons, microglial cells, oligodendrocytes and endothelial cells. They are crucial to brain homeostasis, including trophic support to neurons [63], recycling of neurotransmitters [64], and formation and maintenance of the blood–brain barrier (BBB) [65]. Impairment in their essential cellular functions may lead to a reduced ability to maintain a healthy environment for neighboring neurons. Following different pathological stimuli, such as infection, ischemia, neurodegenerative disorders and aging, astrocytes undergo a pronounced transformation called ‘reactive astrocytosis’, classically characterized by both structural and biochemical changes such as increased levels of glial fibrillary acidic protein and alterations in their immunocompetent capacity. In this regard, the presence of neurotoxic reactive astrocytes has been detected in postmortem brain from several neurodegenerative diseases including PD, suggesting that astrocytes could play a critical role in these complex neurological diseases [42, 55].

Human astrocytes are significantly different than rodent astrocytes, being larger, more complex and diverse [62, 66]. Astrocytes are derived from late NSC that transit from a neurogenic to a gliogenic phenotype. The molecular nature of the gliogenic switch has remained elusive, and its timing varies across species, from 7 days in the mouse to 6–9 months in humans [67]. These species-specific differences are reflected in methods for in vitro differentiation of pluripotent stems cells, with the initial derivation methods of human astrocytes from iPSC requiring 3–6 months [68–70]. Several protocols have been optimized to generate in less than 3 months mature human astrocytes from iPSC and seem to follow four main steps: (1) conversion of iPSC to rosette-forming neuroepithelial cells; (2) regional patterning of NSC; (3) specification of the glia lineage by long-term expansion of NSC; and (4) terminal differentiation and maturation of astrocytes [71–75]. Recently, protocols based on the forced expression of cell-type-specific transcription factors such as NF1B, NFA1 and SOX9 have been found to accelerate this process to 3–7 weeks, and to produce functionally mature human astrocytes [76–78].

Recent studies using astrocytes generated from iPSC examined the contribution of astrocytes to PD pathogenesis. Du et al. applied a co-culture system of iPSC-derived neural progenitor cells and astrocytes showing the ability of astrocytes to rescue differentiation defects and mitochondrial dysfunction in iPSC-derived DAn upon rotenone or KCN [79], highlighting an important role of astrocytes in preserving mitochondrial homeostasis of DAn. In addition to this study, astrocytes generated from patients carrying the LRRK2-G2019S mutation, exhibited dysfunctional protein degradation pathways leading to a progressive α-syn accumulation inside the mutant astrocytes. Importantly, using a neuron-astrocyte co-culture approach, we also described for the first time that α-syn is transferred from astrocytes to surrounding DAn affecting their survival [80] (Table 1). Chemical activation of chaperone-mediated autophagy partially restored normal α-syn activity and rescued the neurodegenerative phenotype observed in DAn cultured with PD astrocytes. However, the incomplete restoration suggests that other factors other than α-syn are being secreted by PD astrocytes, and thus contributing to trigger DAn cell death. Future studies may thus be needed to examine whether PD astrocytes carrying LRRK2 mutation have defects in mitochondrial or ER-related pathways like DAn, and test their contribution to inflammatory-dependent alterations. In contrast to these studies, Booth and colleagues focused on genes involved in astrocytic neuroprotective capacity and found a downregulation of TGFB1 and matrix metallopeptidase 2 (MMP2), in LRRK2-G2019S mutated astrocytes [81]. Finally, astrocytes carrying GBA1 mutation showed increased hypertrophy and expression of astrocytic GFAP and S100β as compared to controls. After exposing them with α-syn, GBA astrocytes substantially increased the secretion of inflammatory cytokines and accumulated α-syn aggregates [82], thus suggesting that astrocytes may play a role in α-syn accumulation and processing, contributing to neuroinflammation.

## Human iPSC-derived microglia

Microglial cells are the resident immune cells of the CNS, representing 5–20% of the cell population. They provide the first line of defense for the innate immune system by their rapid activation upon infection or injury [83], migrating to the injured region and secreting several pro-inflammatory cytokines to eliminate invading pathogens. They also release trophic and anti-inflammatory factors to enhance neuronal survival and regeneration.

As the primary CNS immune cells, microglia are highly responsive and react very rapidly to neuroinflammation. In response to a variety of stimuli, including α-syn, microglia can become activated and initiate an inflammatory response in the CNS. Recent genetic studies have highlighted the importance of these cells in several neurodegenerative disorders including AD, ALS and PD. Also, microglia activation has been reported in PD patient brains and in living patients, according to PD postmortem studies [44, 49, 84]. Interestingly GWAS studies revealed an association between Crohn’s disease or leprosy and LRRK2 mutations, suggesting a possible link between *LRRK2* and inflammatory diseases [85, 86]. Moreover, LRRK2 expression levels are increased in immune cells in PD [87]. LRRK2 deficiency or inhibition in microglial cells attenuates inflammation upon stimulation with lipopolysaccharide (LPS) [88, 89], pre-formed α-syn fibrils [90] or HIV-Tat protein [91], whereas the opposite inflammatory effect is observed by its overexpression, pointing to a positive inflammatory regulation of this gene in those cells [92, 93]. Other well-known PD-related risk genes such as *PINK1* and *PARK7* are also related to inflammatory-dependent processes. *PINK1*-deficient mice show enhanced TNF-α, IL-6 and IL-1β production over time, leading to increased neuron death [94], and *PARK7*-deficient microglia show an increase in inflammatory mediators and reactive oxygen species (ROS) [95].

Although it has been shown that microglia are directly involved in the regulation of the immune response in the brain of rodent models, whether microglial activation in humans produces detrimental or beneficial effects in surrounding DAn is largely unknown.

Recently, several groups have reported the differentiation of human iPSC-derived microglia-like cells, allowing the investigation of the role of genes involved in neurodegeneration in the myeloid lineage [96–99]. The main differences between these studies lie in how progenitor cells were obtained, and the manner in which they further mature into microglial-like cells. These differences include: (1) formation of embryoid bodies [96]; (2) the use of hypoxia culture conditions [98]; and (3) cell sorting protocols for CD43+  [98], CD34+ /CD43+  [97], CD14+ or CD14+ /CX3CR1+  [99] cells, to increase the purity of the progenitor population. In all studies, progenitors were differentiated directly with the addition of specific factors that boost microglial fate, such as IL-3, IL-34, GM-CSF and/or M-CSF [96, 97, 99], TGF-β1, CX3CL1 and CD200 [98], or were co-cultured with neurons [100] or astrocytes [97], reducing the time of differentiation. Ultimately, these protocols generated microglia-like cells that express the human microglia gene signature [101], are positive for specific microglia markers by flow cytometry and immunofluorescence, and are functional in terms of phagocytosis, ADP/ATP response and cytokine production.

In the context of immune cell behavior, several studies have highlighted significant differences in monocytes/macrophages from PD patient-derived iPSC that could contribute to pathophysiology. Speidel and colleagues demonstrated that the LRRK2-G2019S mutation accelerated the differentiation of iPSC-derived monocytes compared with non-mutant isogenic controls, and also increased pro-inflammatory release of TNF-α upon LPS stimulation, with deficits in migration capacity [102]. Also, Haenseler et al. derived macrophages from iPSC of patients with the *SNCA* A53T mutation and SNCA triplication, and found increased levels of intracellular α-syn, which compromised phagocytosis as determined by measuring fibrillar α-syn uptake [100] (Table 1).

Although the concept of neuroinflammation in PD has been principally attributed to microglia, the innate immune cells of the CNS, recent studies have showed an activation of T-lymphocytes by α-syn, and T-cell subset alterations in blood samples of PD patients, suggesting a possible role of adaptive immune cells in PD pathogenesis [103–105]. In addition, a recent study by Sommer et al. applied a co-culture system between T-lymphocytes and iPSC-derived midbrain neurons to investigate the role of T-cells in sporadic PD. Of interest, the autologous co-culture of patient midbrain neurons with patient IL-17 producing T-cells leads to increased neuronal cell death compared to a non-autologous co-culture. This effect was dependent on IL-17 receptor signaling, as blockage of IL-17 or IL-17 receptor rescued neuronal death [43].

## Human iPSC-derived endothelial cells and pericytes

The BBB comprises a multicellular neurovascular unit where pericytes, astrocytes, and neurons are all in direct contact with brain microvascular endothelial cells (BMECs). In turn, BMECs form a specialized transport barrier created by tight junctions and polarized efflux pumps, and this finely tuned cellular architecture permits the blood-to-brain passage of crucial nutrients and metabolic molecules while prohibiting the transport of deleterious factors and most drugs [106]. It has recently become clear that physiological aging and pathological neurodegeneration are associated with a prominent impairment in BBB permeability, structure and functional integrity. These events are closely linked to endothelial dysfunction in cerebral microvessels, deregulation of endothelial–pericyte–astroglial communications, changes in neuronal excitability and glia-controlled support of neuronal activity, and deregulation of angiogenesis. These mechanisms might serve as a basis for maintaining long-lasting neuroinflammation, and for ineffective or incorrect action of drugs whose action requires overcoming the BBB.

The homeostatic control of the endothelial phenotype resides in cells in close proximity to the vessels such as astrocytes, pericytes, neurons and microglia. Thus, interactions between these cells will shape many of the properties of the brain endothelium and affect the BBB. In fact, activation of microglia and reactive astrocytes leads to the release of pro-inflammatory cytokines, including IL-6, IL-1β, and TNF-α, and ROS, which can induce neuronal death and rearrange tight junction protein expression on endothelial cells, leading to alterations in the BBB [107].

Although it was initially assumed that the BBB was unaffected in PD, several studies have reported an increase in BBB permeability in midbrain and striatum in patients [108, 109]. In addition to human studies, several toxin-induced animal models present a disruption in the BBB that is associated with DAn loss [110]. Whether altered neuroinflammatory mechanisms in PD may compromise BBB functionality, rendering the CNS more vulnerable to neurotoxic substances and immune cells from the periphery, remains unknown. Importantly, there are striking differences in BBB substrate specificity and transporter expression and activity between humans and animals [111], thus hampering our understanding of their contribution to human neurodegenerative diseases.

iPSCs offer an unprecedented opportunity for in vitro BBB modeling through the generation of human BMECs. Moreover, the advantage of iPSCs to generate different cell types such as astrocytes, pericytes, neurons and microglia, will allow investigating the homeostatic control of the brain endothelium. iPSC-derived BMECs have been used either alone, in combination with iPSC-derived neural cells and astrocytes, or together with rodent primary pericytes and astrocytes [112, 113]. However, while pericyte differentiation protocols exist, no BBB model incorporating all iPSC-derived endothelial cells, pericytes and astrocytes has yet been reported.

In the context of PD, iPSC-derived BMECs from a preclinical patient carrying compound heterozygous loss-of-function mutations in *PARK2* associated with familial early-onset PD failed to show active P-glycoprotein in apical-to-basolateral transport assays, which may indicate that patients with familial PD mutations are predisposed to loss of P-glycoprotein function [114]. However, these results were very preliminary and would need to be rigorously confirmed across multiple iPSC lines from different patients harboring the same mutation. Thus, future studies should aim to co-culture BMECs with astrocytes and pericytes to dissect the specific contribution of those diverse cell types to the BBB properties in PD.

## Human iPSC-derived oligodendrocytes

The primary function of oligodendrocytes is the generation of the myelin sheath, to insulate axons and organize the distribution of voltage-gated ion channels required for the proper conduction of action potentials. In addition, oligodendrocytes provide trophic support to nerve axons and can mediate inflammation [115]. Thus, oligodendrocytes play a key role in myelin-related diseases, including multiple sclerosis, leukodystrophies, and periventricular leukomalacia, and an increasing awareness of their potential role in neurodegenerative disease is recently emerging [116].

In the context of PD, while it is primarily considered a gray matter disease, recent investigations suggest that alterations in white matter may accompany or even play a role in the disease process [117]. Noteworthy, an integration of several GWAS resulted in the identification of oligodendrocytes as genetically associated with PD, confirming an alteration in those cells at the earliest stages of disease progression [60]. Thus far, the role of oligodendrocytes in the pathogenesis of PD remains elusive.

Protocols for derivation of oligodendrocytes from iPSC have been developed, but they require long culture periods (70–150 days) and they show limited efficiency [118–120]. Recently, the induction of transcription factors SOX10, OLIG2 and NKX6.2 in iPSC-derived neural progenitors has improved oligodendrocyte differentiation considerably, resulting in up to 70% of O4+ oligodendrocytes within 28 days. Induction of oligodendrocytes and myelination in 3D organoid models has also recently been reported [121, 122] and may provide a valuable platform in deciphering the role of oligodendrocytes in PD pathogenesis.

Mutations and multiplications in *SNCA* have been identified in some cases of familial PD, and these patients occasionally present with oligodendroglial inclusions positive for α-syn [123]. Moreover, transgenic mice overexpressing human α-syn under different oligodendrocyte-specific promoters develop DAn degeneration [124, 125]. Although the mechanism by which α-syn accumulates in oligodendrocytes is unknown, it could result from the uptake of α-syn released from neurons. To investigate this issue, Djelloul and colleagues derived oligodendrocytes from human iPSC of multiple system atrophy (MSA), a neurological disorder with synucleinopathy-related degeneration in the nigrostriatal dopamine system and PD, both containing α-syn inclusions, and demonstrated the endogenous expression of α-syn in oligodendrocyte lineage cells [126].

## Organoids and in vivo systems

Although the co-culture of iPSC-derived glia cells with neurons can be used to recreate disease pathology in vitro allowing for deeper research into the cross-talk pathogenesis of the disease, they overlook the spatial organization of the microenvironment that may confer physiological properties. To overcome this limitation, recent progress in the generation of three-dimensional (3D) brain organoids, which consists of complex multiple region-specific cell types mimicking an organ-like structure [127], have allowed to create more accurate and representative models of neurodegenerative diseases. As a step towards modeling PD in brain organoids, novel protocols were used to generate human organoids containing DAn with midbrain identity [128, 129]. Interestingly, it has been reported that iPSCs or patterned NSCs, can be aggregated, starting from single cells, to form human midbrain organoids. After 2 months from their generation they contain functional TH-positive DAn expressing specific midbrain markers, such as Forkhead box protein A2 (FOXA2), or dopamine transporter (DAT). Moreover, DAn from midbrain organoids not only showed electrophysiological activity but they also accumulated neuromelanin inside their cytoplasm, thus offering an opportunity for studying the role of this pigment in the disease process which remains largely underexplored [129].

Of interest, disease modeling of PD using LRRK2-mutated PD iPSC-derived 3D midbrain organoids, has revealed a decrease in DAn, increased aggregation of α-syn and impaired neurite complexity [130, 131]. In addition, the use of 3D-iPSC models has recently provided a new platform to investigate the chronic effects of drug treatment in sporadic PD and to study the environmental contributors to disease pathogenesis in a more realistic complex system [132]. 3D-based neuronal culture models have been shown to innately develop not only astrocytes [133–135] but also microglial cells [136] and oligodendrocytes in a spontaneous manner [121, 122, 137, 138]. Such 3D models have been recently developed to study PD under neurotoxic MPTP conditions including neurons, astrocytes and oligodendrocytes [139] but also adding microglia and BBB [140]. These 3D co-culture models provide the opportunity to study the interaction between different cell types to create a simplified “brain in a dish”.

Although all these studies are very promising, brain organoids are currently very poorly reproducible models due to the stochastic generation of neural tissue that depends on the self-organization and self-development capacities of the tissue, and the random differentiation of the diverse neuronal types in the organoid. New reproducible protocols and supportive scaffolds would advance the development of controlled large tissue constructs to generate different spatially separated brain region identities to study the influence of brain connections such as nigrostriatal and cortico-striatal projections; in addition to examine the pathological process in PD progression from the dorsal motor nucleus in an ascending fashion where midbrain and forebrain structures are subsequently affected.

An additional strategy to study iPSC-derived brain cell phenotypes and cell–cell interactions is their transplantation into the brain of living rodents, thus allowing to overcome the limitation represented by the fact that the cells are maintained in culture dishes in an artificial environment. Therefore, the incorporation of human iPSC-derived brain cells in rat brain or human non-primates represent an important step towards modeling PD in an in vivo environment, and offer a potential tool for regenerative therapy [141, 142]. The technical and ethical obstacles of iPSC treatment in humans may limit the feasibility of transplanting reprogrammed stem cells, but this opportunity in PD treatment is unprecedented [143].

To avoid immunogenic rejection of the graft, host strains must lack an adaptive immune system, as in athymic nude mice, severely compromised immunodeficient (SCID) mice or non-obese diabetic (NOD)/SCID humanized mice [144]. Xenografting of human iPSCs into mice, has been performed with iPSC-derived neurons [141, 145, 146] and microglia [147], as well as with whole 3D organoids that exhibit functional integration with in vivo neural circuits and vasculature [148]. These xenoculture systems open a window to study in vivo cell-type interactions using patient cells during aging and neurodegeneration. In addition, transplantation of stem cells from patients in whom the genetic cause is known will allow to perform mechanistic studies to elucidate the interactions between their key genes and how they impact the function of cells in the brain.

## Conclusions

**Fig. 2:**
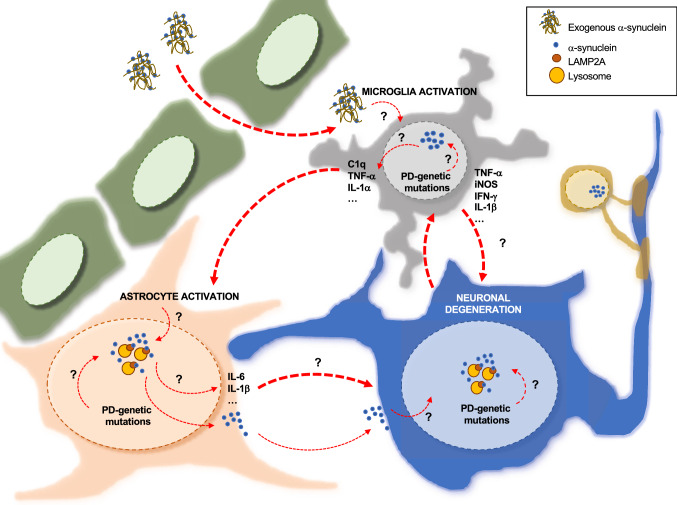
Schematic representation of α-syn-induced Parkinson’s disease pathogenesis in the central nervous system**.** Neuron = blue; astrocyte = orange; microglia = gray; oligodendrocyte = yellow; endothelial cells = green. It has been postulated that increased permeability of the blood–brain barrier (BBB) in Parkinson’s disease (PD) might allow the transit of neurotoxic substances and α-syn from the periphery to the central nervous system. Exogenous α-syn activates microglia, which leads to the secretion of several pro-inflammatory cytokines. These secreted molecules might, on the one hand, activate astrocytes to become reactive and secrete other neuroprotective and neurotoxic cytokines affecting neuronal survival. On the other hand, microglial-secreted molecules might hamper neuronal survival itself. Importantly, PD-associated genetic mutations contribute to this neurotoxic loop by inducing the intrinsic accumulation of α-syn in astrocytes, microglia and neurons, which might in turn affect cellular functionality

Unfortunately, much of the existing research in studying the human nervous system at the molecular level has always been challenging due to the complexity of the brain, and the difficulty of obtaining live human neurons in the laboratory. The advent of iPSC technology enables human somatic cells to be reprogrammed to pluripotency, and viable human cells affected by the disease to be generated. During the last years, several laboratories have validated this new technology for gaining new perspectives on neurodegenerative pathological mechanisms within PD-DAn. However, PD is a complex disease that involves not only neurons but also other CNS cell types, as seen by the emerging notion of neuroinflammation occurring in PD. It is noteworthy how non-neuronal cells have gained attention in the past recent years switching PD research focus into a non-neuro-centric view (Fig. 2). Co-cultures of different cell types differentiated from PD patients and control iPSCs will allow the identification of non-cell-autonomous disease mechanisms and biomarkers that could predate DAn neurodegeneration. Yet, there is a concern as to how well iPSC-derived cells can model late-onset diseases such as PD, where patients do not develop symptoms until later in life, implicating age as a necessary component to disease progression. Several iPSC studies have demonstrated a loss of particular age-associated features during iPSC induction including telomere length, mitochondrial fitness and loss of senescence markers in iPSCs derived from old donors, suggesting that rejuvenation takes place during reprogramming [149, 150]. In this sense, several protocols are emerging to improve cell differentiation to enhance their maturity state and reflect their brain regional-dependent diversity that may affect their function. In this regard, complex experimental models including tissue-specific organoids, and mouse brain hybrids are promising tools to overcome these issues. However, there is still a need for further improvement with supportive scaffolds that may help to control the development of large tissue constructs. Initial set-up studies have emerged for 3D modeling of PD in which different brain cell types are combined. However, the majority of them use the well-known dopaminergic neurotoxic MPP+ and rotenone as model compounds. Thus, new organoid systems for different genetic variants associated with PD should be studied.

In relation to future co-culture techniques, the use of iPSC-derived glia cells and neurons could help recreating disease pathology, allowing a better understanding of the gene networks that underlie the neuroprotective roles of astrocytes for example, and how these networks are perturbed in chronic disease states, but also to explore the role of glia cells in a healthy context. Such disease models are the first of their kind, and could also be valuable for the discovery of new drug candidates for PD. Finally, by studying symptomatic and asymptomatic mutation carriers, iPSC technology could also provide a unique opportunity for identifying putative gene-linked PD biomarkers in pre-symptomatic individuals, opening a new window into early diagnosis and individualized treatment of the preclinical phase of the disease.
